# The characteristics and factors of the internalizing and externalizing behaviours of children at high risk for autism spectrum disorder

**DOI:** 10.1186/s12888-021-03479-6

**Published:** 2021-10-22

**Authors:** Ning Ding, Huiyun Gao, Jiying Jiang, Mengyao Zhai, Huan Shao, Linyan Fu, Chunyan Li, Yanling Ren, Yu Li, Min Feng, Xiwen Cui, Nana Qiu, Peiying Jin, Xiaoyan Ke

**Affiliations:** Nanjing Brain Hospital, Affiliated with Nanjing Medical University, Child Mental Health Research Center, Nanjing, 210029 China

**Keywords:** Autism spectrum disorder, Parent-child interactions, Parenting stress, Internalizing behaviour, Externalizing behaviour

## Abstract

**Background:**

The behavioral characteristics of children with autism spectrum disorder (ASD) are not only affected by their disease, but also by their parenting environment. HR-ASD has the risk of developing internalization and externalization problems. How the early development of these behavioral problems is affected by parent-child interaction is worth exploring. We tested whether parent-child interactions and parenting characteristics were associated with behavioural problems during the infant periods.

**Methods:**

This study collected data from 91 infants at high risk for ASD and 68 matched typically developing (TD) infants, about their internalizing and externalizing behavioural problems and engagement states (i.e. positive, negative, and parent-child interactions), using free play paradigm. Parent measures were assessed using the Broad Autism Phenotypic Questionnaire (BAPQ) and Parenting Stress Index Short Form (PSI-SF) questionnaire. The core symptoms of ASD were assessed using the the Autism Diagnostic Observational Schedule (ADOS).

**Results:**

During free play, infants in the HR-ASD group showed more internalizing (*P* < 0.001) and externalizing (*P* < 0.05) behaviours and less positive engagement (*P* < 0.01) than the TD group. In the regression analysis, we found that parenting stress had an impact on the infants’ externalizing behaviours (△R^2^ = 0.215). Parent negative engagement had an impact on the infants’ internalizing behaviours (△R^2^ = 0.451).

**Conclusions:**

The present study revealed that children at high risk for ASD exhibited more severe internalizing and externalizing behavioural problems than TD group. The parent negative engagement is associated with behavioural problems. The findings on the contribution of parents’ factors to behavioural problems suggests that the parenting stress and parent-child interactions are important factors for mitigating behavioural problems.

## Background

Autism spectrum disorder (ASD) is a developmental disability marked by early-onset social communication deficits and repetitive sensory-motor behaviours [1–4]. The comorbidity of psychiatric symptoms in children with ASD is receiving increasing attention in the literature, such as behavioural disorders and anxiety disorders [5–7]. Emotional and behavioural problems in ASD are frequent, and rates of attention deficit-hyperactivity disorder, social anxiety, and oppositional disorder are elevated than typically developing children [8–10]. Specifically, the development of internalizing and externalizing behaviors has risen to high priority in behavioural neuroscience [11, 12]. Emotional and behavioural problems (EBPs), including internalizing behaviours (e.g., social dysfunction, anxiety, depression, cognitive problems and avoidance symptoms) as well as externalizing behaviours (e.g., impaired concentration, hyperactivity, aggression and behavioural disorders) [4, 13–15]. For internalizing behaviours, Carter et al [16] found that children with autism reported an increase in social anxiety as they grew older, whereas typically developing children reported a decrease in social and evaluative anxiety as they grew older [17]. Additionally, for externalizing behaviors, several studies have reported that children with ASD have more severe externalizing behaviors such as poor attention, disruptive, hyperactive, delinquent and aggressive behaviors than typically developing children [18–21]. However, the literature so far has mainly focused on older children with ASD, and few studies have focused on behavioral problems in high-risk autistic infants, and their findings are mixed.

Previous studies mainly explored how autistic traits influence individuals’ social and behavioural development, such as children with ASD are generally considered to exhibit less positive engagement, more negative engagement [22], and a lower ability to express emotionality compared with their typically developing peers, which results in their EBPs. Besides, daily management of children with ASD is a major challenge for their parents, particularly during early childhood, and there is a finding suggesting that the experiences of a positive home environment might be sufficient for a sharp decline in externalizing problems [17]. The behaviours of children with ASD may create challenges for caregivers due to the disorder’s interference with help-seeking behaviours, which affects the long-term prognosis [23, 24]. As revealed from parents’ recollections of their children’s behaviours before diagnosis and from analyses of family videos, early abnormal development occurs during the first 1–2 years of life. This abnormal development manifests as extreme passiveness, obvious irritability [25, 26], and a lack of response to parental voices, attempts to play and interactions [27, 28].

Recent research suggests that infants’ regulatory problems can be best understood in a relational context and that disturbances of parent-child interactions and parenting stress are significant risk factors for infants’ EBPs [29, 30]. These studies evaluating parental stress suggested that the severity of autistic traits could also be related to parenting stress and highlighted the daily management of behavioural problems and children’s characteristics (age, adaptive behaviours, and gender) as relevant sources of parenting stress [3, 31–33]. Early research showed that parenting and family interaction variables explained up to 30 to 40% of children behavioural problems [34]. Positive parenting (involving praise, encouragement, and affection) are strongly associated with children’s positive engagement, high child self-esteem and interaction tendency and are protective against later behavioural problems and comorbidity [35]. Many family correlates of aggressive child behaviours are present in infancy before the onset of such coercive cycles [36]. Although internalizing and externalizing problems are well recognized in the mental health profiles of children with ASD, information on the role of their parents and, particularly in infancy, is scarce [37, 38]. Children’s internalizing and externalizing behaviours make their parents more likely to experience negative emotionality, and the consequent increase in mutual negative emotionality reduces interactions, thereby aggravating behavioural problems [39]. It is of great importance to identify children at risk for high and continuous internalizing and externalizing problems early in development [17, 40].

The current study had three aims. First, compare the level of child and parent engagement states, parent-child interactions and behavioural problems. Second, the correlation analysis was conducted to determine the association between behavioural problems, child engagement state and parent engagement state. Third, a hierarchical multiple regression analysis was performed to predict the proportion of variance in the behavioural problems that could correspond to age, parenting stress, parent negative engagement, and parent-child interactions.

## Methods

### Participants

The participants were members of the Children’s Mental Health Research Center of the Nanjing Brain Hospital affiliated with Nanjing Medical University from October 2017 to February 2020 who were invited to participate in a cohort study. A total of 159 children participated in this study. The sample included 91 infants at high risk for autism (70 boys; age range, 8–30 months) and 68 TD infants (40 boys, age range, 8–30 months).

*The ASD at-risk group* were selected on a clinical assessment by two child psychiatrists based on of the following: i) positive M-CHAT assessment results [41], ii) scores above 30 on the Childhood Autism Rating Scale (CARS) [42], iii) all cases satisfied either risk criteria for ASD (if under 24 months of age at intake) or DSM-5 criteria for ASD diagnosis [43] if over 24 months of age at intake, and received both iv) the Autism Diagnostic Observational Schedule (ADOS) [44], and v) the Autism Diagnostic Interview-Revised (ADI-R) [45].

*The TD group* included 68 infants (40 boys; age range, 8–30 months) and their parents. The TD infants had no neuropsychiatric disorders and were matched with the HR-ASD group on mental age and the ratio of boys to girls. TD participants were screened for ASD using the M-CHAT and CARS. The groups were matched on the raw scores of the Gesell Developmental Assessment (DQ), a standardized test assessing IQ in children aged 6 months and older. The DQ scores in the TD group were higher than 80. The exclusion criteria were as follows: any genetic syndromes or neurological conditions; a history of craniocerebral trauma; chronic medical conditions; or visual, hearing or motor impairments.

### Ethical considerations

All parents signed an informed consent form. Ethical approval for the study was granted by the China Clinical Trial Registration Center (Name of the Ethic Committee: Nanjing Brain Hospital Ethics Committee), ChiCTR-OPC-17011995. The study was approved the Nanjing Brain Hospital Ethics Committee (Approval certificate number: 2017-KY098–01).

### Procedure

#### Diagnostic and cognitive assessment

All children were screened at enrolment with the M-CHAT, the Autism Behaviour Checklist (ABC [46]) and the CARS [42]. The ABC is a well-established parent-report checklist used to screen for and diagnose autism. The Gesell Developmental Schedules assess five domains of cognitive abilities: adaptive behaviour, gross motor behaviour, language behaviour, fine motor behaviour and personal-social behaviour [47]. The TD children were screened at enrolment. The average raw scores of three of the domains (adaptive behaviour, gross motor behaviour and fine motor behaviour) were above 80 in both groups; there were group differences in the scores of the other two domains (language behaviour and personal-social behaviour).

#### Parent measures

The Broad Autism Phenotypic Questionnaire (BAPQ) assesses the possibility that the personality and language characteristics. The BAPQ is a 36-item self-report questionnaire designed to measure BAP characteristics in relatives of individuals with ASD. Each question is rated on a 6-point scale, ranging from 1 (i.e., very rarely) to 6 (i.e., very often) as to how often the individual experiences each item. The total score is calculated by averaging all 36 items and higher scores indicate high BAP traits [48].

The Parenting Stress Index Short Form (PSI-SF) is a self-report questionnaire with 36 items used to evaluate parenting stress on a 5-point Likert-type scale. The PSI-SF consists of three subscales, parenting distress (PD), difficult child (DC) and parent-child dysfunctional interaction (PCDI), as well as a total stress scale [49, 50].

#### Parent-child free play

Parent-child interactions were videotaped during a semi-structured 15-min play session in a room built for the participants at the study site in the Children’s Center of Nanjing Brain Hospital. First, children played with 3 toys of the parent’s choice for the first 5 min. Then, children played with their mother or father with a standard set of toys for the following 10 min, as they would at home (as described in National Institute of Child Health and Human Development - Early Childcare and Youth Development NICHD-ECCRN [21]).

#### Coding

##### Free play paradigm

All children underwent behavioural observation during free play. The NICHD Research Handbook of Early Child Care [21, 51–53] was used to measure parent-child interactions. All parenting codes were rated on a global 3-point scale, ranging from 1 (not at all characteristic) to 3 (highly characteristic). *Internalizing and externalizing behaviours.* Internalizing and externalizing behaviours were measured based on the 6 factors of the Child Behaviour Checklist for 2- to 3-year-old children [54–56] and in terms of 7 syndromes (i.e., emotional responses, anxiety/depression, physical complaints, loneliness, sleep problems, attention problems and aggressive behaviours) and 5-DSM orientations (i.e., depression, anxiety, loneliness spectrum, attention deficit hyperactivity disorder, confrontation and aggression). Parent and child behaviours were coded every 10 s with the Noldus Observer 12.0 XT software (Noldus Inc., Netherlands).

The codes were as follows: *child positive engagement*, which included the child’s positive or neutral affect, vocalizations, or positive body posture; *child negative engagement*, which included the child’s impatience, anger, distressed vocalizations and negative body posture, crying, or pushing the parent away; *child withdrawal*, which referred to the child’s withdrawal from a joint activity by expressing sadness or anxiety; *child object exploration*, which referred to the child’s independent exploration of toys without interaction with the parent; *parent positive engagement*, which included the child’s expression of positive or neutral affect, vocalizations and behaviours, or smiling while playing games; *parent negative engagement*, which included the parent’s angry, hostile, irritable, or negative vocalizations; *parent intrusiveness*, which referred to the parent’s dominance, physical manipulation, hostility, or criticism; *parent limit setting*, which referred to the parent’s persistent effort to engage the child, appropriate construction of interaction, or warm limit setting; *dyadic reciprocity*, which included the parent’s or child’s mutual adaptation to the other’s state, give-and-receive reciprocity, or fluent and rhythmic interactions; *internalizing behaviours*, which referred to the child’s disobedience, defiance of orders, reluctance to speak, shyness or timidity, or blank staring; and *externalizing behaviours*, which included the child screaming, stomping, crying, throwing a tantrum, letting himself or herself fall suddenly, making a gesture of hitting, grabbing, knocking his or her head against a wall, tearing clothes up and ignoring calls from others. The free play paradigm was independently coded by two trained research assistants. The interrater reliability for participation status among parents and children was 90.79 and 92.10%, respectively. Any differences in code specifications were resolved through discussion.

### Statistical analysis

The data analyses were conducted using the statistical package SPSS for Windows, version 24. Demographic characteristics were investigated using the chi-squared test for independence (sex) and an independent sample t-test (age, IQ and parent factors such as PSI and BAPQ scores). Statistical significance was defined as a two-sided *p*-value less than 0.05. A Pearson bivariate correlation analysis was conducted to determine the association between child engagement state and parent engagement state.

To identify predictors contributing to the presence of behavioral disorder, a hierarchical multiple regression analysis was performed to predict the proportion of variance in the dependent variable (behavioural problems) that could correspond to the independent variables (age, PSI score, parent negative engagement, and parent-child interactions (dyadic reciprocity)). Blocks of predictors were entered into the model in four steps. The model (model 1) included age as an independent variable and as a control variable for the subsequent analyses. The PSI score was added to model 2, parent negative engagement was added to model 3, and parent-child interactions (dyadic reciprocity) were added to model 4. The increase in variance (△R2) was assessed for each block.

## Results

### Demographic characteristics

The descriptive statistics for all study variables are presented in Table 1. As a preliminary measure to identify developmental covariates for subsequent multivariate analyses, bivariate correlations were conducted between all study variables and developmental scores (age, language behaviour, personal-social behaviour, BAPQ and PSI) for each group. We found no difference in sex between the two groups (x^2^ = 0.366, *P* = 0.235). The DQs for language behaviour and personal-social behaviour from the Gesell Developmental Schedules differed significantly between the two groups (*P* < 0.001), but those for adaptive behaviour, gross motor behaviour and fine motor behaviour did not differ between the HR-ASD group and the TD group. The average BAPQ score in the HR-ASD group was 111.54 (± 12.102). The developmental assessment showed that the children’s average language score was 53.78 (± 21.574) and that the social contact score was 71.14 (± 18.093).

**Table 1:** Demographic and Clinical Characteristics of the Study Participants

Variable	HR-ASD group ***(N*** = 91)		TD group (***N*** = 68)		***t/x***^***2***^
	***M***	***SD***	***M***	***SD***	
*Child measures*					
Age (months)	18.20	8.45	16.11	5.34	1.754
Sex					0.366
*Female*	21		20		
*Male*	70		48		
Gesell (DQ)					
*Language behaviour*	53.78	21.57	92.51	10.76	−13.502**
*Personal-social behaviour*	71.14	18.09	96.73	9.36	−14.806**
ADOS	12.03	3.91	N/S		
*Parent measures*					
BAPQ	111.54	12.10	98.14	17.23	5.727**
PSI	77.82	15.18	66.91	17.04	4.255**

*P < 0.05; **P < 0 .01; ***P < 0.001

### Behaviours during Parent-child interactions

The means and SDs of all interactive variables for the two groups of children and their parents appear in Table 2.

**Table 2:** Parent, Child and Behavioural Problems in the HR- ASD and TD Groups

Variable	HR-ASD group		TD group			
	***M***	***SD***	***M***	***SD***	***t***	***Effect size***
Child positive engagement	15.18	5.68	21.85	4.47	−7.816**	1.287
Child negative engagement	4.62	5.87	2.71	2.01	2.933*	0.440
Child withdrawal	3.87	3.05	5.21	2.25	−3.050**	0.500
Child object exploration	7.57	4.57	4.19	1.78	5.779***	0.975
Externalizing behaviours	2.96	4.76	1.85	1.17	1.866*	0.317
Internalizing behaviours	4.21	3.11	2.72	1.44	3.663***	0.415
Parent positive engagement	19.35	5.39	21.60	5.04	−2.676**	0.431
Parent negative engagement	4.02	3.47	2.40	1.80	3.522*	0.586
Parent intrusiveness	4.34	1.69	2.87	1.17	6.170***	1.011
Parent limit setting	6.40	4.82	6.63	4.58	−0.224	0.049
Dyadic reciprocity	11.90	6.14	15.99	5.92	−4.215***	0.678

*P < 0.05; **P < 0.01; *** *P* < 0.001

### Parent interactive behaviour

Using independent-sample t-tests, we examined group (HR-ASD and TD) and parent differences in the following parental behaviours: positive engagement, negative engagement, intrusiveness, and limit setting. The results revealed a main effect for parent intrusiveness, *P* < 0.001, ES = 1.011. The independent-sample t-tests indicated that TD parents showed appropriate negative engagement (*P* < 0.05) and greater positive engagement (*P* < 0.01) compared with ASD parents.

### Child interactive behaviour

The independent-sample t-tests of child behaviours including positive engagement, negative engagement, withdrawal and child object exploration yielded no significant group effect. Children with ASD exhibited less positive engagement (*P* < 0.001), more negative engagement (*P* < 0.01), more withdrawal (*P* < 0.01), and more child object exploration (*P* < 0.001) compared with the TD group. The HR-ASD group also exhibited more internalizing behaviours (*P* < 0.01) and more externalizing behaviours (*P* < 0.01) than TD group.

### Dyadic reciprocity

The independent-sample t-test showed a main effect for group, *P* < 0.001, ES = 0.678. TD children and parents were observed to be more reciprocal compared with the HR-ASD group.

### Correlations between child factors and Parent factors

The simple correlations and summary statistics for all variables are presented in Table 3. Parent negative engagement and the PSI score were found to be related to several measures of child negative engagement and child behavioural problems (ranging from 0.205 to 0.833, *P* < 0.05). Parent-child interactions were correlated with more behavioural problems during free play in children at high for risk ASD. Parent positive engagement was strongly correlated with child positive engagement (r^2^ = 0.831, *P* < 0.05), and child negative engagement showed weaker but nonetheless substantial correlations with the other parenting variables. Less parent negative engagement, less child negative engagement and greater parent positive engagement were associated with fewer internalizing behaviours and externalizing behaviours. Parent negative engagement was correlated with internalizing behaviours (r^2^ = 0.205, *P* < 0.05) and with externalizing behaviours (r^2^ = 0.252, *P* < 0.05). Greater parent-child interactions were correlated with child positive engagement (r^2^ = 0.833, *P* < 0.05) and parent positive engagement (r^2^ = 0.753, *P* < 0.05). Finally, child negative engagement was correlated with a higher PSI score (r^2^ = 0.210, *P* < 0.05), parent negative engagement during free play (r^2^ = 0.426, *P* < 0.05), and more child externalizing behaviours (r^2^ = 0.442, *P* < 0.05).

**Table 3:** Bivariate Associations Between the Main Study Variables and Behaviours Problems

Variable	Age	PSI	BAPQ	C-pos	C-negative	P-pos	P-neg	Inter-	Exter-	D-rec
**Age**	–									
**PSI**	−0.037	–								
**BAPQ**	−0.019	− 0.067	–							
**C-pos**	−0.044	− 0.068	0.043	–						
**C-neg**	−0.177	0.210*	−0.151	− 0.420**	–					
**P-pos**	0.031	−0.108	0.093	0.831**	−0.312**	–				
**P-neg**	0.059	0.426**	−0.015	− 0.324**	0.352**	− 0.253*	–			
**Inter-**	0.120	0.103	−0.065	− 0.346**	0.071	− 0.379**	0.205*	–		
**Exter-**	0.121	0.147	−0.110	−0.468**	0.442**	−0.399**	0.252*	0.560**	–	
**D-rec**	−0.052	− 0.078	0.129	0.833**	−0.359**	0.753**	−0.357**	− 0.322**	− 0.483**	–
**P-limit**	−0.092	0.118	0.078	0.001	0.110	−0.010	0.166	−0.126	−0.067	− 0.108

0.2 < r < 0.4 low correlations, 0.4 < r < 0.6 moderate correlations, 0.6 < r < 0.8 high correlations

*C-pos* Child positive engagement, *C-neg* Child negative engagement, *P-pos* Parent positive engagement, *P-neg* Parent negative engagement, *Inter-* Internalizing behaviours, *Exter-* Externalizing behaviours, *D-rec* Dyadic reciprocity, *P-limit* Parent limit setting

**P < 0.01; *P < 0 .05

### Regression of Parent factors on Behavioural problems

A hierarchical multiple regression analysis was performed to predict the proportion of variance in the dependent variable (internalizing behaviours) that could be attributed to the independent variables (PSI score, parent negative engagement, and dyadic reciprocity). The results from the hierarchical regression are presented in Table 4. The results when the PSI score was entered into the age model were as follows: △R^2^ = 0.013, △F = 1.169, *P* = 0.283. Adding parent negative engagement into the model significantly improved model fit (△R^2^ = 0.451, △F = 75.457, *P* < 0.001). Finally, the inclusion of dyadic reciprocity did not significantly improve model fit (△R^2^ = 0.002, △F = 0.268, *P* = 0.606). The results from the hierarchical regression on externalizing behaviours are presented in Table 5. Adding the PSI score to the age model in significantly improved model fit (△R^2^ = 0.215, △F = 24.078, *P* < 0.001). The model fit also significantly improved when parent negative engagement was entered into the model (△R^2^ = 0.071, △F = 8.755, *P* = 0.004). Finally, the inclusion of dyadic reciprocity moderately improved the model (△R^2^ = 0.128, △F = 18.755, *P* < 0.001).

**Table 4:** Summary of the Hierarchical Regression Analysis of Child Internalizing Behaviours

Variable	Model 1		Model 2		Model 3		Model 4	
	***B***	***t***	***B***	***t***	***B***	***t***	***B***	***t***
Constant	4.258	4.607***	1.935	0.828	0.355	0.205	0.854	0.430
Age	0.054	1.173	0.063	1.351	0.050	1.464	0.050	1.445
PSI			0.028	1.081	0.014	0.726	0.013	0.682
P-negative					0.721	8.687	0.696	7.261
D-reciprocity							−.0280	−0.518
R2	0.015		0.028		0.480		0.481	
F	1.376		1.274		26.720***		19.939***	
△R2			0.013		0.451		0.002	
△F			1.169		75.457***		0.268	

The table displays the unstandardized regression coefficients for the four models. *P < 0.05. **P < 0.01. ***P < 0.001. *P-negative* Parent negative engagement, *D-reciprocity* Dyadic reciprocity

**Table 5:** Summary of the Hierarchical Regression Analysis of Child Externalizing Behaviours

Variable	Model 1		Model 2		Model 3		Model 4	
	***B***	***t***	***B***	***t***	***B***	***t***	***B***	***t***
Constant	6.620	4.087***	−9.862	−2.698	−10.954	−3.107*	−3.245	−0.883
Age	−0.027	− 0.332	0.038	0.522	0.029	0.419	0.023	0.356
PSI		3.008	0.196	4.907	0.186	4.848	0.175	4.979
P-negative					0.498	2.947	0.118	0.668
D-reciprocity							−0.434	−4.331
R2	0.001		0.216		0.287		0.415	
F	0.110		12.108***		11.673**		15.230***	
△R2			0.215		0.071		0.128	
△F			24.078***		8.755**		18.755***	

The table displays the unstandardized regression coefficients for the four models. *P-negative* Parent negative engagement, *D-reciprocity* Dyadic reciprocity. *P < 0.05. **P < 0.01, ***P < 0.001

## Discussion

As in previous research [8, 10, 15], the present study identified high levels of emotional and behavioural problems in infants at high risk for ASD. The results suggested that children’s engagement states were influenced by their parents’ characteristics. No age effect was found on internalizing or externalizing problems for infants with and without ASD. For the HR-ASD group, more parenting stress was associated with child negative engagement and parent negative engagement. Child negative engagement was associated with externalizing problems and parent negative engagement. Positive child engagement was associated with parent-child interactions, externalizing and internalizing behaviours. These results contribute to the growing body of work suggesting that at a very early age in children’s development, the level of parent-child interactions shows significant development and already shows links to parent (e.g., parenting stress, parent negative engagement) and individual characteristics and factors.

### Link to Parent-child interactions

This study suggests that a critical factor of externalizing behaviours is negative participation, indicating that an increase in negative participation among children may lead to externalizing behaviours, thereby increasing the risk of future comorbidities. Some autistic children with negative emotionality show destructive behaviours (tantrums or physical attacks in anger), while others may interact in a way that interferes with their target behaviours (excessive agitation or depression) [57]. Negative participation among children at high risk for ASD (e.g., frowning, refusing free play and crying in free play) may be initially attributed to their increasing emotional response and poor emotional control. Poor emotional regulation can be inherent in autistic children [58], leading to externalizing behaviours (e.g., throwing objects, crying and stomping). Second, negative emotionality is also considered to be associated with autistic children’s “alexithymia”, which is “the difficulty in identifying, describing and distinguishing emotionality”. Research by Costa suggests that teaching children with ASD how to identify and distinguish their emotionality from that of others by improving their “alexithymia” may be useful for regulating their emotionality [59]. The critical factor of internalizing behaviours is child object exploration. Internalizing behaviours predict more emotional problems (e.g., anxiety and depression) in the future. Children’s object exploration affects internalizing behaviours because children at high risk for ASD engage in less active socialization and prefer to be immersed in their own worlds. ASD is an early-onset complex neurodevelopmental disorder that affects two aspects of children’s emotional lives: their emotionality is primarily negative [60], and their ability to express emotionality is weak. Notably, infants with ASD also have less positive emotionality than TD children. Children’s fear is a self-protective characteristic that is attributed to their responses to threatening or unfamiliar stimuli, and an increase in fear among infants predicts subsequent behavioural inhibition and anxiety [61]. The research on temperament in the context of ASD suggested that children with ASD may be associated with an overall profile of lower surgency-extraversion and higher negative affectivity [62, 63]. The rise of negative affectivity predicts subsequent externalizing behaviours [60]. Furthermore, some studies demonstrate that parents can help regulate stress hormones and brain activity in children, but not adolescents [56].

Given the well-documented challenges of raising children with ASD, research identifying factors that appropriate parent-child interactions and parents engagement state may help reduce behavioural problems. Synchronous interaction through child feeding can buffer the impact of adverse environments on child outcomes, making sensitive care an important target for interventions with high-risk families. Parent-child interactions may help improve variations in the volume of brain area (e.g., the amygdala of ASD children), which in turn improves sociability and alleviates the excessive growth of the amygdala at an early stage [64]. Caregivers often vigorously adjust young children’s behaviour and physiology (by reducing the stress response attributed to the amygdala and the involvement of the frontal lobes) but allow behavioural and physiological development to unfold naturally during puberty. It has been proposed that there are sensitive periods before puberty. Early in the life of a child, behavioural interventions can be used to regulate the child’s mind and physiology, thereby offering early life programming of the most optimized brain structure, which is vital to lifelong emotionality. Thus, children’s development can be supported to gradually reduce subsequent developmental complications.

### Links to parenting stress

Pressure in a family is more likely to make parents become sensitive caregivers, and it mediates many adverse child outcomes associated with emotional regulation and behavioural problems [65–67]. As expected, we found that parenting-related stress and relationship variables was higher in parents of infants with ASD than in the TD group. Our findings also suggested that the parenting-related stress was associated with behavioural problems in infants with ASD. This replicates the findings of Weitlauf AS et al. [68] using cross-sectional regression analysis to examine that relationship quality and parenting stress may also represent important factors for young children with ASD. In the hierarchical regression analysis, consistent with previous literature, children’s behavioural problems, particularly externalizing behaviours, were found to appropriately contribute to the parenting stress of parents of infants with ASD [23]. Family factors, particularly parenting stress are also likely important determinants above and beyond child factors [68]. Our findings are consistent with previous studies of parents of preschool children with ASD, but few studies have examined infants aged approximately 8–30 months. Future studies are required to better understand how emotional and societal factors contribute to parenting stress in families of infants with ASD. Parenting demands change as children develop, so further research examining the developmental trajectory of the relationships between parenting stress and problem behaviours throughout the lifespan is necessary. Such future studies will improve our understanding of the role of infant behaviours, parent-infant interactions, and temperament in behavioural development and emotional regulation in the first years of life.

### Limitations

The present study has limitations. First, no more parent variables, such as education and family adversity, were explored. Second, a crosssectional design was utilized while a longitudinal approach might help to determine whether the behavioural problems identified in this study improve as reduced parenting stress and improved parent engagement states. Our research group evaluated and predicted children’s emotions and behaviours problems after 2 years, which will be reflected in the following articles.

## Conclusions

The present study discussed more internalizing and externalizing behavioural problems in infants at high risk for ASD than for typically developing peers. The parent negative engagement is associated with externalizing and internalizing behaviours. The findings on the contribution of parents’ factors to behavioural problems suggests that the parent-child interactions are an important factor for behavioural problems. The amelioration of parenting stress is associated with externalizing behaviours. The results suggested that parents could pay more attention to the behavioural problems of infants at high risk for ASD and adjust parenting stress and dyadic reciprocity appropriately.

## Data Availability

The datasets used or analysed during the current study available from the corresponding author (Xiaoyan Ke) on reasonable request.

## Abbreviations

ASD: Autism spectrum disorder
EBPs: Emotional and Behavioural Problems
TD: Typically Developing
ADOS: Autism Diagnostic Observational Schedule
ADI-R: Autism Diagnostic Interview-Revised
CARS: Childhood Autism Rating Scale
DQ: Developmental Assessment
ABC: Autism Behaviour Checklist
BAPQ: The Broad Autism Phenotype Questionnaire
PSI-SF: Parenting Stress Index Short Form
PD: Parenting Distress
DC: Difficult Child
PCDI: Parent-child Dysfunctional Interaction
NICHD-ECCRN: National Institute of Child Health and Human Development – Early Childcare and Youth Development
